# Insulin lispro low mixture twice daily vs basal insulin glargine once daily and prandial insulin lispro once daily as insulin intensification strategies in patients with type 2 diabetes: Latin American subpopulation analysis of a randomized trial

**DOI:** 10.1186/s13098-016-0163-3

**Published:** 2016-09-17

**Authors:** Arturo Rojas, Georgina Sposetti, Jorge L. Gross, Douglas Eugenio Barbieri, Ran Duan, Bruno Linetzky, Janaina Martins De Lana, Oded Stempa, Angel Rodriguez

**Affiliations:** 1 Direccion-investigacion, Servicio Medico Nutricional S.C. (Centro de Diabetes de Coatzacoalcos), Revolucion # 522, 96400 Coatzacoalcos, VER México; 2 Diabetes and Metabolism Research Department, Head en Instituto de Investigaciones Clínicas, Mar del Plata, Argentina; 3 Centro de Pesquisas em Diabetes, Porto Alegre, Rio Grande do Sul Brazil; 4 Eli Lilly do Brasil Ltda, São Paulo, Brazil; 5 Eli Lilly, Indianapolis, USA; 6 Eli Lilly Interamerica Inc., Buenos Aires, Argentina; 7 Eli Lilly and Co, México City, México; 8 Lilly Spain, Alcobendas, Spain

**Keywords:** Diabetes mellitus, type 2, Insulin intensification, Insulin lispro, Insulin lispro mixture, Latin America

## Abstract

**Background:**

This post hoc analysis examined the efficacy and safety of twice-daily insulin lispro low mixture (LM25) and once-daily basal insulin glargine plus once-daily prandial insulin lispro (IGL) in a Latin American subpopulation with type 2 diabetes mellitus (T2DM).

**Methods:**

A phase 4, randomized, open-label, parallel-arm trial included participants aged 18–75 years with T2DM taking once-daily insulin glargine and stable doses of metformin and/or pioglitazone with glycated hemoglobin (HbA1c) 7.5–10.5 % and fasting plasma glucose ≤121 mg/dL. Participants were randomized 1:1 to receive their stable dose of metformin and/or pioglitazone plus twice-daily LM25 or IGL for 24 weeks. The primary efficacy outcome was change in HbA1c after 24 weeks of treatment. Results from participants in Argentina, Brazil, and Mexico are presented here.

**Results:**

162 participants (80 LM25; 82 IGL) with mean ± standard deviation (SD) age = 57.3 ± 9.0 years and body mass index = 31.3 ± 5.2 kg/m^2^ were included. Mean ± SD change in HbA1c from baseline to week 24 was −1.5 ± 1.0 % (LM25) and −1.1 ± 1.2 % (IGL). At week 24, 35.1 % (LM25) and 31.6 % (IGL) of participants achieved HbA1c <7.0 %. Mean ± SD weight gain from baseline to week 24 was 2.4 ± 2.9 kg in the LM25 group and 1.0 ± 3.1 kg in the IGL group. The mean ± SD rates of total hypoglycemia per year were 18.9 ± 27.3 (LM25) and 21.6 ± 31.1 (IGL). Rates of treatment-emergent adverse events were 46 % (LM25) and 39 % (IGL).

**Conclusions:**

Our results suggest that both LM25 and IGL are viable treatment options for insulin intensification in Latin American patients with T2DM with suboptimal glycemic control on basal insulin glargine. The safety and tolerability profiles of LM25 and IGL are consistent between this Latin American population and the global trial-level population.

*Trial registration* NCT01175824

## Background

Type 2 diabetes mellitus (T2DM) is an important health problem in Latin America, particularly in Brazil and Mexico, which respectively have the fourth and sixth largest populations of people with diabetes in the world [1]. In 2014, the prevalence of T2DM in South and Central America was 8.1 % (24.8 million patients) [2] and is projected to increase by 60 % (to 38.5 million patients) by 2035 [1]. This increase is partly caused by changes in diet and lifestyle, e.g., urbanization, an increase in the consumption of animal products and processed foods, and increases in patient body mass [3–5]. Moreover, only 36 % of patients with T2DM in Latin America and only 26 % in Brazil have a glycated hemoglobin level (HbA1c) at the recommended level of <7.0 % [6, 7]. Considering the changing lifestyle and the increasing prevalence of T2DM in the region, current scientific evidence on insulin intensification strategies from global populations must be verified in a Latin American population.

As T2DM progresses, there is a decline in insulin secretory capacity such that, for most patients, treatment with insulin must be initiated to achieve the target HbA1c ≤7 % [8]. The Latin American Diabetes Association (ALAD) guidelines recommend initiating insulin therapy with a long-acting basal insulin analogue, such as insulin glargine, in combination with oral agents for patients who fail to achieve target HbA1c on oral agents alone [3]. Unfortunately, the long-term efficacy of basal insulin therapy alone is often limited [9], with less than 50 % of patients reaching target HbA1c, commonly due to excessive postprandial glycemic excursions [10]. Therefore, most patients with T2DM will require an intensification of their insulin therapy.

The ALAD guidelines recommend intensifying insulin therapy with a combination of 2 types of insulin [3]. To this end, there are 2 possible strategies for intensifying insulin therapy: switching to a premixed combination of long-acting and short-acting insulin administered twice daily [3, 10] or continuing on basal insulin and adding rapid-acting insulin before meals [3, 11]. There are few data directly comparing these 2 insulin intensification strategies in Latin American patients with T2DM inadequately controlled by basal insulin plus oral antidiabetic agents. This post hoc analysis of a multinational clinical trial compared the efficacy and safety of twice-daily insulin lispro low mixture (LM25) and once-daily basal insulin glargine plus once-daily prandial insulin lispro (IGL) in Latin American patients with T2DM who had not achieved target HbA1c on once-daily basal insulin glargine with metformin and/or pioglitazone.

## Methods

### Study design

This study was a post hoc analysis of a subpopulation of Latin American participants from a multinational, randomized, open-label, noninferiority, phase 4 clinical trial designed to examine the efficacy and safety of 2 insulin intensification strategies in patients with T2DM not adequately controlled on once-daily basal insulin glargine with metformin and/or pioglitazone [12]. The global study was approved by an independent ethical review board at each study site and was conducted in accordance with the Declaration of Helsinki, the International Conference on Harmonisation Good Clinical Practice standards, and all local laws and regulations in the study countries. The trial was registered with ClinicalTrials.gov: NCT01175824. All participants provided written informed consent.

### Study population

In the global study, participants were enrolled at 55 study sites in Argentina, Brazil, China, Egypt, India, Republic of Korea, México, Romania, Russian Federation, Spain, and Turkey. This post hoc analysis included data from participants who were enrolled in Latin America (Argentina, Brazil, and Mexico).

The inclusion criteria were: age 18–75 years; a diagnosis of T2DM consistent with the World Health Organization Classification of Diabetes [13]; HbA1c 7.5–10.5 %; current regimen of stable doses of metformin (≥1500 mg/day for at least 8 weeks) and/or pioglitazone (≥30 mg/day for at least 12 weeks); a current stable regimen of once-daily basal insulin glargine for at least 90 days before screening; and fasting plasma glucose ≤121 mg/dL, determined by the central laboratory, or >121 mg/dL if the investigator determined further titration of basal insulin glargine was not possible for safety reasons. Exclusion criteria were: a screening body mass index >45 kg/m^2^; more than 1 severe hypoglycemic episode within 24 weeks before screening; and a history of using drugs contraindicated for use with the study drugs.

At the screening visit, demographic and clinical data were collected from all participants. Participants also underwent a physical examination and provided a fasting plasma glucose (FPG) sample for measurement in a central laboratory.

### Treatment protocol

The trial treatment protocol has been described in detail previously [12]. Briefly, participants were randomized to receive subcutaneous twice-daily LM25 (insulin lispro low mixture; 75 % insulin lispro protamine suspension and 25 % insulin lispro solution) or IGL (once-daily basal insulin glargine and once-daily prandial insulin lispro), in addition to their stable dose of metformin and/or pioglitazone, for 24 weeks. LM25 was administered before breakfast and dinner. Insulin glargine was administered at bedtime. Insulin lispro was administered before the largest meal of the day. The largest meal of the day was defined as the meal with the highest 2-h postprandial blood glucose concentration and was determined during the screening period. LM25, insulin glargine, and insulin lispro were administered using 100 U/mL prefilled pens.

### Outcome measures

The primary efficacy endpoint was the change in HbA1c from baseline to week 24. The secondary efficacy endpoints were the percentage of participants reaching the HbA1c target levels <7.0 and ≤6.5 %, the change in FPG concentration from baseline to week 24, 7-point self-monitoring of blood glucose (SMBG) profiles at baseline and week 24, glycemic variability at week 24 [as measured by the standard deviation (SD)] in 7-point SMBG profiles, daily total, basal, and prandial insulin doses at week 24, and change in body weight at week 24.

Safety endpoints included treatment-emergent adverse events (TEAEs), and the incidence, rate, and severity of hypoglycemic episodes. In addition, participant satisfaction with insulin treatment was measured using the insulin treatment satisfaction questionnaire (ITSQ) [14]. Participant perceptions about the acceptability and effectiveness of diabetes medications and perceived adverse effects were measured using the perceptions about medications-diabetes 21 (PAM-D21) questionnaire [15]. Total scores on the ITSQ range from 0 to 100, where 100 indicates complete satisfaction with insulin treatment. Subscale scores on the PAM-D21 range from 0 to 100, where higher scores indicate better perceptions about diabetes medications.

### Statistical analysis

The intent-to-treat (ITT) and safety populations were both defined as all randomized participants who received at least 1 dose of study drug. Efficacy and health outcome endpoints were analyzed using the ITT population. Summary statistics were calculated by treatment group for all endpoints. Due to the relatively small sample size, no statistical comparisons were made between treatment groups.

## Results

### Participant disposition

**Fig. 1:**
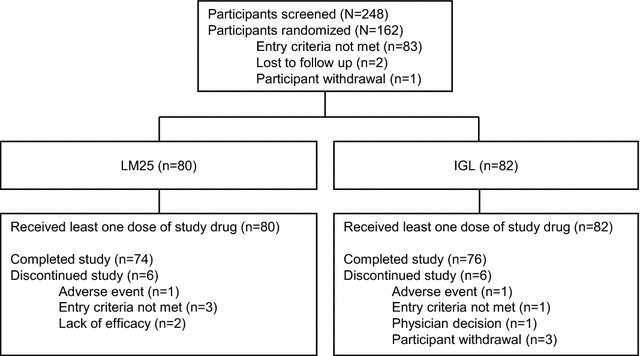
Participant disposition. Summary of participant disposition of Latin American participants with type 2 diabetes mellitus who were treated with LM25 or IGL for 24 weeks. *IGL* once-daily basal insulin glargine plus once-daily prandial insulin lispro, *LM25* 75 % insulin lispro protamine suspension and 25 % insulin lispro solution

A total of 248 patients were screened for study entry in Latin America. Of these, 162 were eligible for inclusion in the study and received at least 1 dose of study drug, 12 discontinued, and 150 completed the study (Fig. 1).

### Baseline characteristics

Baseline demographic and clinical characteristics are summarized in Table 1.

**Table 1:** Demographic and baseline characteristics

Characteristic	LM25 (N = 80)	IGL (N = 82)	Total (N = 162)
Country, n (%)			
Argentina	40 (50.0)	39 (47.6)	79 (48.8)
Brazil	20 (25.0)	23 (28.0)	43 (26.5)
Mexico	20 (25.0)	20 (24.4)	40 (24.7)
Sex, n (%)			
Male	39 (48.8)	30 (36.6)	69 (42.6)
Female	41 (51.3)	52 (63.4)	93 (57.4)
Age (years), mean (SD)	57.5 (9.7)	57.1 (8.4)	57.3 (9.0)
Weight (kg), mean (SD)	82.5 (15.4	81.8 (15.0)	82.2 (15.1)
BMI (kg/m^2^), mean (SD)	30.9 (4.8)	31.7 (5.5)	31.3 (5.2)
Duration of diabetes (years), mean (SD)	13.8 (7.9)	12.9 (6.8)	13.4 (7.3)
HbA1c at screening			
%, mean (SD)	8.8 (0.8)	8.6 (0.8)	8.7 (0.8)
<8.5 %, n (%)	33 (41.3)	35 (42.7)	68 (42.0)
FPG (mg/dL), mean (SD)	107.1 (37.1)	98.5 (28.2)	102.8 (33.0)
Insulin glargine dose at screening (IU), mean (SD)	39.3 (19.3)	39.5 (18.9)	39.4 (19.0)
Glycemic variability (mg/dL), mean (SD)	46.6 (18.8)	49.7 (18.6)	48.2 (18.7)
Concomitant oral antidiabetic drugs			
Metformin, n (%)	80 (100)	82 (100)	162 (100)
Daily dose (mg), mean (SD)	1968.1 (393.0)	2062.8 (396.8)	2016.0 (396.6)
Pioglitazone, n (%)	4 (5.0)	5 (6.1)	9 (5.6)
Daily dose (mg), mean (SD)	30.0 (0.0)	30.0 (0.0)	30.0 (0.0)
Metformin and pioglitazone, n (%)	4 (5.0)	5 (6.1)	9 (5.6)

*BMI* body mass index, *FPG* fasting plasma glucose, *HbA1c* glycated hemoglobin, *IGL* once-daily basal insulin glargine plus once-daily prandial insulin lispro, *IU* international units, *LM25* 75 % insulin lispro protamine suspension and 25 % insulin lispro solution, *SD* standard deviation

### Efficacy

**Fig. 2:**
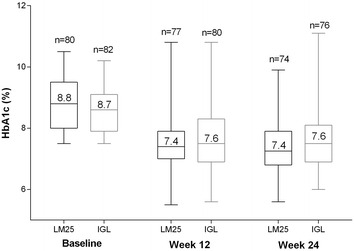
HbA1c levels in participants receiving LM25 or IGL. Observed HbA1c levels at baseline, week 12, and week 24 in participants receiving LM25 or IGL. Values in *boxes* denote mean HbA1c. *HbA1c* glycated hemoglobin, *IGL* once-daily basal insulin glargine plus once-daily prandial insulin lispro, *LM25* 75 % insulin lispro protamine suspension and 25 % insulin lispro solution

With respect to the primary outcome, the mean ± SD change in HbA1c from baseline to week 24 was −1.5 ± 1.0 % in the LM25 group and −1.1 ± 1.2 % in the IGL group. The mean ± SD change in HbA1c from baseline to week 12 was −1.4 ± 1.0 % in the LM25 group and −1.1 ± 1.1 % in the IGL group. The observed HbA1c levels throughout the study are presented in Fig. 2.

A total of 35.1 % (26/74) of participants in the LM25 group and 31.6 % (24/76) of participants in the IGL group achieved HbA1c ≤7.0 %. A total of 14.9 % (11/74) of participants in the LM25 group and 15.8 % (12/76) of participants in the IGL group achieved HbA1c ≤6.5 %.

The mean ± SD FPG at week 24 was 125.5 ± 42.9 mg/dL in the LM25 group and 121.0 ± 39.9 mg/dL in the IGL group. The mean ± SD change in FPG from baseline to week 24 was 17.6 ± 55.2 mg/dL in the LM25 group and 21.6 ± 46.9 mg/dL in the IGL group. The mean ± SD glycemic variability at baseline was 46.6 ± 18.8 in the LM25 group and 49.7 ± 18.6 in the IGL group. The mean ± SD changes in glycemic variability from baseline to week 24 were −9.0 ± 17.3 mg/dL (LM25) and −10.4 ± 17.9 mg/dL (IGL).

**Fig. 3:**
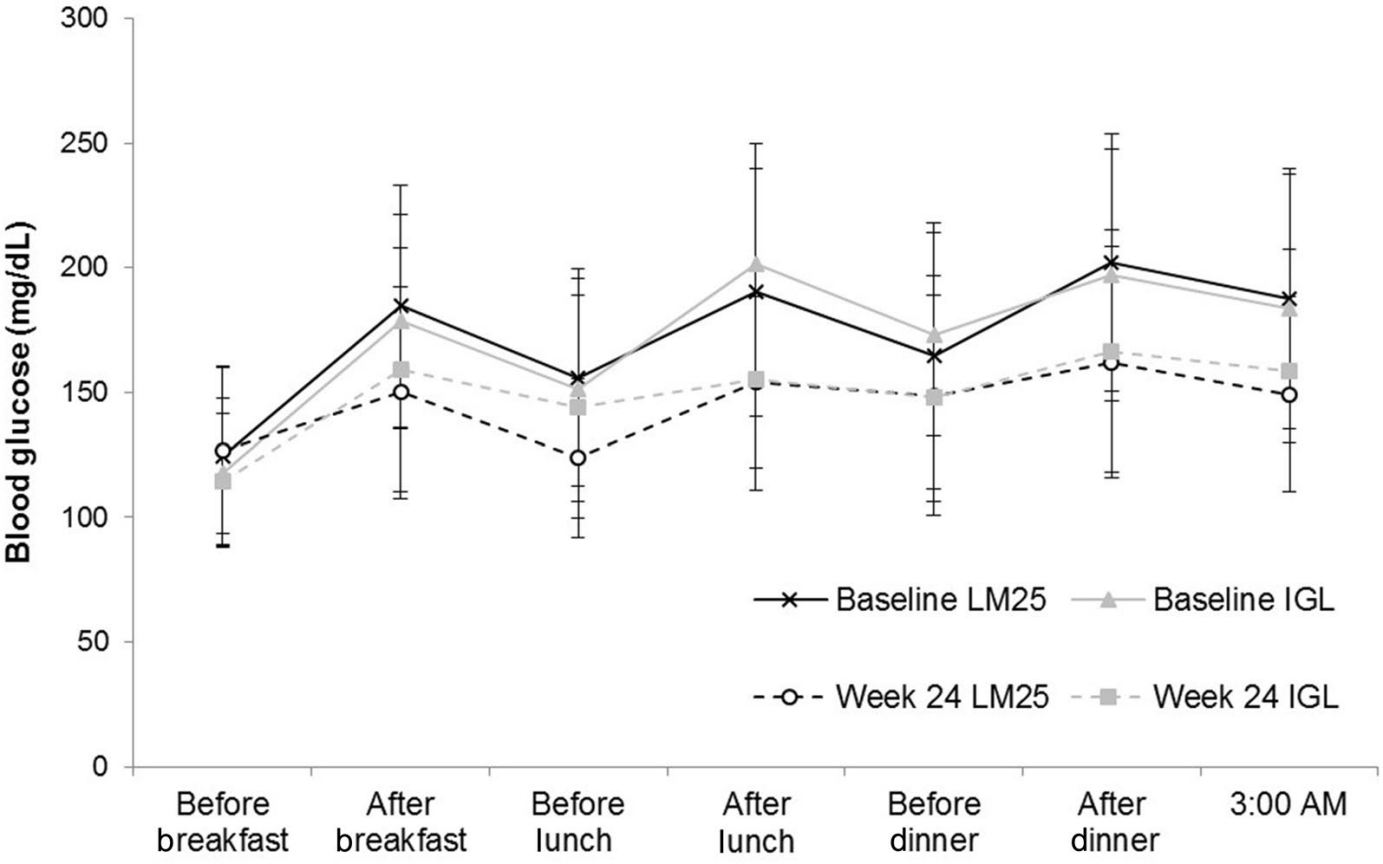
7-point SMBG levels at baseline and week 24. Mean unadjusted 7-point SMBG levels at baseline and week 24 in Latin American participants with type 2 diabetes mellitus who were treated with LM25 or IGL for 24 weeks. *Error bars* indicate standard deviation. *IGL* once-daily basal insulin glargine plus once-daily prandial insulin lispro, *LM25* 75 % insulin lispro protamine suspension and 25 % insulin lispro solution, *SMBG* self-monitoring of blood glucose

The mean unadjusted 7-point SMBG levels at baseline and week 24 are presented in Fig. 3.

Total daily, basal, and prandial insulin doses at baseline and week 24 are presented in Table 2. Of the patients in the IGL group, 10 received prandial insulin lispro at breakfast, 40 at lunch, and 32 at dinner.

**Table 2:** Total daily, basal, and prandial insulin doses at baseline and week 24

Insulin dose	LM25 (N = 80)	IGL (N = 82)
Total daily insulin dose (IU), mean (SD)		
Baseline^a^	40.0 (19.2)	43.9 (18.8)
Week 24	61.0 (27.6)	60.6 (24.3)
Daily basal insulin dose (IU), mean (SD)		
Baseline^a^	30.0 (14.4)	39.8 (18.8)
Week 24	45.8 (20.7)	46.3 (20.2)
Daily prandial insulin dose (IU), mean (SD)		
Baseline^a^	10.0 (4.8)	4.2 (0.8)
Week 24	15.3 (6.9)	14.4 (6.6)

*IGL* once-daily basal insulin glargine plus once-daily prandial insulin lispro, *IU* international units, *LM25* 75 % insulin lispro protamine suspension and 25 % insulin lispro solution, *SD* standard deviation

^a^Baseline in this table is defined as day 1 post-randomization

Participants in both treatment groups experienced weight gain. The mean ± SD changes in body weight from baseline to week 24 were 2.4 ± 2.9 kg (LM25) and 1.0 ± 3.1 kg (IGL).

### Safety and tolerability

At least 1 TEAE was reported by 46 % (37/80) of participants in the LM25 group and 39 % (32/82) of participants in the IGL group. Of these, 7.5 % (6/80) of participants in the LM25 group and 4.9 % (4/82) of participants in the IGL group reported TEAEs that were considered to possibly be related to the study drugs. Serious TEAEs were reported by 3.8 % (3/80) of participants in the LM25 group and 3.7 % (3/82) of participants in the IGL group. One participant in each group discontinued due to adverse events and no participants died during the study.

Overall, approximately 70 % (113/162) of participants experienced at least 1 episode of hypoglycemia during the study (Table 3). Severe hypoglycemia was experienced by 1.3 % (1/80) of participants in the LM25 group and no participants in the IGL group.

**Table 3:** Reported hypoglycemia in study participants

Hypoglycemia	LM25 (N = 80)		IGL (N = 82)	
	Participants with ≥1 episode, n (%)	Number of episodes per participant year, mean (SD)	Participants with ≥1 episode, n (%)	Number of episodes per participant year, mean (SD)
Overall (≤70 mg/dL)	57 (71.3)	18.9 (27.3)	56 (68.3)	21.6 (31.1)
Documented symptomatic (≤70 mg/dL)	45 (56.3)	9.6 (15.5)	40 (48.8)	11.0 (19.4)
Asymptomatic (≤70 mg/dL)	36 (45.0)	8.5 (17.9)	43 (52.4)	10.2 (20.9)
Nocturnal	24 (30.0)	2.4 (5.8)	22 (26.8)	2.8 (6.5)
Severe	1 (1.3)	0.1 (0.7)	0 (0)	0 (0)

*IGL* once-daily basal insulin glargine plus once-daily prandial insulin lispro, *LM25* 75 % insulin lispro protamine suspension and 25 % insulin lispro solution, *SD* standard deviation

### Health outcomes

The mean changes in ITSQ and PAM-D21 from baseline to week 24 are presented in Table 4.

**Table 4:** Changes in ITSQ and PAM-D21 questionnaire scores

Variable, change from baseline	LM25 (N = 80)		IGL (N = 82)	
	Baseline	Change from baseline	Baseline	Change from baseline
ITSQ, mean (SD)				
Inconvenience of regimen	92.7 (10.7)	−1.5 (11.0)	92.2 (12.1)	0.5 (11.2)
Lifestyle flexibility	82.9 (21.2)	−0.1 (24.1)	84.7 (17.6)	−4.8 (24.9)
Glycemic control	80.9 (19.9)	6.9 (20.0)	78.3 (21.7)	10.3 (22.1)
Hypoglycemic control	85.5 (14.7)	−2.7 (17.7)	85.7 (17.8)	0.1 (17.0)
Insulin delivery device satisfaction	86.9 (15.7)	4.2 (16.1)	88.6 (14.3)	1.1 (10.6)
Total score	86.5 (11.4)	1.1 (11.6)	86.8 (12.4)	1.2 (10.6)
PAM-D21, mean (SD)				
Convenience/flexibility	90.0 (16.0)	−0.7 (21.2)	91.7 (13.8)	−1.2 (16.8)
Perceived effectiveness	74.6 (20.7)	5.9 (23.7)	72.0 (20.5)	12.0 (25.4)
Emotional effects	84.2 (20.2)	1.0 (21.7)	86.8 (17.5)	−0.2 (22.4)
Physical effects	87.0 (13.4)	1.0 (12.8)	90.5 (12.9)	1.1 (10.4)

*IGL* once-daily basal insulin glargine plus once-daily prandial insulin lispro, *ITSQ* insulin treatment satisfaction questionnaire, *LM25* 75 % insulin lispro protamine suspension and 25 % insulin lispro solution, *PAM-D21* perceptions about medications-diabetes 21, *SD* standard deviation

## Discussion

To our knowledge, this is the first report to disclose results from a Latin American subpopulation in a study comparing a twice-daily premixed insulin regimen with a once-daily basal insulin plus once-daily prandial insulin regimen in patients with T2DM not adequately controlled on insulin glargine with metformin and/or pioglitazone. One important aspect to highlight is the inclusion of patients ‘failing’ on insulin glargine plus oral medication, defined as FPG <121 mg/dL with high HbA1c, meaning that prandial intensification was likely needed. In these patients, further titration of insulin glargine may increase the risk of hypoglycemia. While we did not make any statistical comparisons in this post hoc analysis due to the limited sample size, numerical improvements in HbA1c after 24 weeks, likely to be clinically relevant, were observed with both LM25 and IGL. Our approach is consistent with the ALAD guidelines for intensifying insulin therapy in patients with HbA1c >7.0 % and our results suggest that both LM25 and IGL can be effective for lowering HbA1c in Latin American patients with T2DM who have blood glucose levels not adequately controlled on oral agents and basal insulin.

The reduction in HbA1c levels after 24 weeks of treatment experienced by both groups in the Latin American subpopulation is consistent with, and numerically higher than, the trial-level results in participants of various countries and ethnicities, which showed LM25 to be noninferior, and subsequently superior, to IGL with respect to glycemic control as measured by the change in HbA1c over the 24-week treatment period [12]. In keeping with the primary efficacy finding, improvements in secondary efficacy outcomes, including the proportion of participants who achieved HbA1c targets and SMBG, were also observed in the 2 study groups in the Latin American subpopulation. The secondary efficacy outcome results are also consistent with the trial-level findings [12].

Our observation that FPG increased and HbA1c decreased from baseline in both treatment groups appears somewhat paradoxical. This phenomenon was numerically greater in the LM25 group. Findings from a previous study suggested that the relative contribution of postprandial glucose to HbA1c increases as glycemic control improves, whereas the contribution of FPG increases as diabetes worsens [16]. Based on FPG, the patients in this study were already optimized on glargine, therefore, the benefit was most likely due to the postprandial component with both intensification strategies.

We observed a trend for numerically lower blood glucose concentrations before lunch in the LM25 group compared with the IGL group. This difference in before-lunch blood glucose was also observed at a numerically lower level in the trial-level results [12]. Interestingly, in another study (DURABLE), Hispanic participants had significantly lower postprandial glucose after breakfast compared with the Caucasian participants [17]. Patients in Latin America vary with respect to the timing of their main daily meal. LM25 was administered before morning and evening meals in both our study and the DURABLE trial, regardless of when the main meal was consumed. Administering LM25 and then consuming a small meal, or a meal with high levels of dietary fiber or a low glycemic index at breakfast time, as is common in Argentina, could account for the lower blood glucose concentrations observed in Hispanic and Latin American participants [18]. Conversely, consuming only breakfast and dinner, as is common in Mexico, may also result in low blood glucose during the day; however, administering LM25 at breakfast and dinner in this situation could be appropriate. Importantly, a recently published subanalysis of the trial-level results demonstrated that glycemic control improved in patients receiving either LM25 or IGL, regardless of the timing of the main daily meal [19].

Despite the lower blood glucose concentrations before lunch with LM25 and the general concern that premixed insulin may increase the risk of hypoglycemia [20], we found that, while the rates of hypoglycemia in the Latin American subpopulation were numerically higher than those observed in the trial-level population [12], both the incidence and rate of hypoglycemia during the study were similar between the 2 study groups. It is important to note that these similar rates of hypoglycemia were observed in the context of a 0.5 % difference in HbA1c. Only 1 participant experienced severe hypoglycemia and this did not result in a discontinuation. The numerically higher overall rate of hypoglycemia may be explained by specific dietary patterns, exercise habits, or cultural practices in the Latin American population. It is also notable that the mean insulin dose was numerically higher in this Latin American population compared with the trial population [12]. This higher dose may have contributed to the numerically higher rates of hypoglycemia observed in this analysis.

We also found that participants in the LM25 group gained more weight over the 24-week treatment period than participants in the IGL group. This trend was also observed in the trial-level results (LM25 = 1.13 kg, IGL = 0.50 kg) [12]; however, the difference between treatments was more pronounced in the Latin American subgroup. One reason for this may be that patients can overeat to avoid hypoglycemia, particularly in cases where a frugal breakfast is eaten, which is often the case in Argentina and Brazil. Our finding of increased weight gain with LM25 should be considered within the broader context of improved glycemic control and the absence of specific recommendation for the management of diet and exercise in the management of weight in patients with T2DM [11]. The numerically higher insulin dose observed in this Latin American subpopulation compared with the trial-level population [12] may also have contributed to the weight gain observed in this analysis.

This subanalysis of a multinational study has a number of strengths, including the prospective, multisite, multicountry, randomized design and the Latin American study population, which permits the exploration of the efficacy of insulin regimens in patients with varied meal patterns. As with most clinical trials of insulin, our study also has a number of limitations including: the post hoc nature of the analysis and open-label design of the study; the fact that the 2 insulin regimens used different injection devices and had different dosing requirements; the fixed distribution of doses at breakfast and dinner, which may have been better given at different times in some regions; and the relatively small sample size in each of the 3 Latin American countries. The study was not powered to compare the ethnic subgroups within the study population and, therefore, no statistical comparisons were made between groups. The differences in prescribing practices, dietary habits, and clinical guidelines among Latin American countries mean that the results of this post hoc analysis may not be generalizable to other Latin American countries.

## Conclusions

Intensification of insulin therapy with either LM25 or IGL improved HbA1c in Latin American patients with T2DM who had not achieved target HbA1c on once-daily basal insulin glargine with metformin and/or pioglitazone. The overall safety profile was similar between groups. The results of this study add to the body of evidence that supports the current ALAD guidelines, which recommend both insulin regimens as an option for insulin intensification in patients who do not achieve target HbA1c on basal insulin with oral antidiabetic drugs.

## Abbreviations

ALAD: Latin American Diabetes Association
FPG: fasting plasma glucose
HbA1c: glycated hemoglobin
IGL: once-daily basal insulin glargine plus once-daily prandial insulin lispro
ITSQ: insulin treatment satisfaction questionnaire
ITT: intent-to-treat
LM25: insulin lispro low mixture; 75 % insulin lispro protamine suspension and 25 % insulin lispro solution
PAM-D21: perceptions about medications-diabetes 21
SD: standard deviation
SMBG: self-monitoring of blood glucose
T2DM: type 2 diabetes mellitus
TEAE: treatment-emergent adverse event
